# Gender differences in the association between self-rated health and hypertension in a Korean adult population

**DOI:** 10.1186/1471-2458-12-135

**Published:** 2012-02-19

**Authors:** Hee-Young Shin, Min-Ho Shin, Jung-Ae Rhee

**Affiliations:** 1 Department of Biomedical Science, Chonnam National University Medical School, Gwangju, Korea; 2 Department of Preventive Medicine, Chonnam National University Medical School, 5 Hak-dong, Dong-gu, Gwangju 501-746, Korea

## Abstract

**Background:**

Self-rated health (SRH) has been reported as a predictor of mortality in previous studies. This study aimed to examine whether SRH is independently associated with hypertension and if there is a gender difference in this association.

**Methods:**

16,956 community dwelling adults aged 20 and over within a defined geographic area participated in this study. Data on SRH, socio-demographic factors (age, gender, marital status, education) and health behaviors (smoking status, alcohol consumption, physical activity) were collected. Body mass index and blood pressure were measured. Logistic regression models were used to determine a relationship between SRH and hypertension.

**Results:**

32.5% of the participants were found to have hypertension. Women were more likely than men to rate their SRH as poor (*p *< 0.001), and the older age groups rated their SRH more negatively in both men and women (*p *< 0.001). While the multivariate-adjusted odds ratio (OR, 95% CI) of participants rating their SRH as very poor for hypertension in men was OR 1.70 (1.13-2.58), that in women was OR 2.83 (1.80-4.44). Interaction between SRH and gender was significant (*p *< 0.001).

**Conclusions:**

SRH was independently associated with hypertension in a Korean adult population. This association was modified by gender.

## Background

Self-rated health (SRH) is based on a simple question where people are asked to rate their own overall health. It is a subjective consciousness about personal health rather than an objective measure of health. However, SRH has been reported as a predictor of morbidity and mortality even after controlling for other related factors [1-8]. Furthermore, recent studies revealed that SRH is associated with inflammatory or immune markers in healthy individuals as well as patients, which support a biological basis of SRH [9-11]. Although the mechanisms are not clear, SRH may be considered both subjective and objective measures of health. Few studies have investigated SRH as an outcome of hypertension, one of common morbidities [12,13], but SRH has not been examined as a risk factor for hypertension.

Gender differences in health vary according to differential vulnerabilities in men and women. Previous studies on gender difference in the relationship between SRH and mortality implied a different process by which men and women assess their general health state [1,7,14-18]. While men are likely to reflect mainly serious and life-threatening disease in their assessment of SRH, women are likely to reflect both life-threatening and non-life-threatening disease [19]. In addition, the accuracy of self-assessment for excellent SRH in different gender and at different age groups may affect the result [20]. Since hypertension is mostly asymptomatic and a chronic health condition, women may rate their health status by reflecting the chronic condition rather than men. Therefore, the association between SRH and hypertension can be modified by gender.

The purpose of this study was to examine whether SRH is independently associated with hypertension and whether the association is modified by gender in a Korean adult population.

## Methods

### Study participants

The study population was community-dwelling men and women aged 20 and over within a defined geographical area of Jeollanamdo Province, South Korea in 2006. A total of 1,485,843 community residents (730,832 men and 755,011 women) were identified from the national registration records and 41,250 candidates were selected by random sampling stratified by cities and counties to be a representative sample of Jeollanamdo Province. In total, 16,956 residents provided informed consent and participated in the Jeollanamdo Community Survey, which collected information on the health behavior of community-dwelling adults. The response rate was 41.1%.

This study relied on secondary data from a Jeollanamdo Community Survey. The data set included the socio-demographic information (age, gender, marital status, education), health behaviors (smoking status, alcohol consumption, physical activity), body mass index, blood pressure and SRH. Only non-identifiable aggregate results were released and they were openly available. No institutional review board approval was sought for this reason.

### Assessments and measurements

Well-trained Health Center staffs visited randomly selected residents. Socio-demographic information (age, gender, marital status, education) and health behaviors (smoking status, alcohol consumption, physical activity) were collected from the participants using structured questionnaires. Smoking status was categorized as current smoker, former smoker, and non-smoker. Alcohol consumption and physical activity were identified as yes or no.

SRH was based on the answer to the question, "in general, how would you describe your current health?" The responses were categorized into five levels: excellent, good, fair, poor or very poor.

Weight and height were measured and body mass index (BMI) was calculated as weight in kilograms divided by height in meters squared. Blood pressure was measured by a mercury sphygmomanometer in the sitting position. The average of two consecutive readings of systolic and diastolic blood pressure was used in the analysis. Participants were considered to have hypertension if the average systolic blood pressure exceeded 140 mmHg and/or their diastolic pressure exceeded 90 mmHg, or they were taking antihypertensive drugs.

### Statistical analysis

*T*-test and chi-square tests were used to examine the distribution of covariates for subjects by hypertension status, and a trend test for age by SRH and chi-square test for gender by SRH were performed. The prevalence of hypertension with 95% confidence intervals was presented by SRH, and a trend test for hypertension by SRH was done. Logistic regression models were used to adjust for other covariates. SRH was entered as a categorical variable, and excellent SRH was taken as a reference group. After finding significantly related factors with hypertension in univariate analysis, age, marital status, education, smoking status, alcohol consumption, physical activity and BMI were entered as covariates in the models. Because of gender differences in the association between SRH and hypertension, regression models were stratified and interaction terms were tested. IBM SPSS statistics 19 software was used for the analysis. A *p*-value of less than 0.05 was considered significant.

## Results

### Prevalence and related factors of hypertension

5,505 (32.5%) of the participants were found to have hypertension. Age, gender, marital status, education, smoking status, alcohol consumption, physical activity, BMI and SRH were identified as the related factors of hypertension by *t*-test or chi-square test (Table 1).

**Table 1:** Characteristics of study participants

Characteristics	No hypertension	Hypertension	*p*-value
	(N = 11,451; 67.5%)	(N = 5,505; 32.5%)	
Age (y), mean (SD)	50.3 (16.8)	63.0 (14.1)	< 0.001
Gender, n (%)			
Men	5,225 (67.5)	2,518 (32.5)	< 0.001
Women	6,226 (67.6)	2,987 (32.4)	

Marital status, n (%)			
Married	8,311 (68.5)	3,816 (31.5)	< 0.001
Others	3,140 (65.0)	1,689 (35.0)	

Education, n (%)			
Elementary school or less	4,166 (54.9)	3,416 (45.1)	< 0.001
Middle or high school	4,287 (73.9)	1,515 (26.1)	
Undergraduate or graduate	2,754 (85.6)	463 (14.4)	

Smoking status, n (%)			
Non-smoker	7,418 (68.5)	3,415 (31.5)	< 0.001
Former smoker	1,180 (57.4)	877 (42.6)	
Current smoker	2,405 (69.5)	1,053 (30.5)	

Alcohol consumption, n (%)			
No	7,204 (68.8)	3,266 (31.2)	< 0.001
Yes	4,029 (65.3)	2,143 (34.7)	

Physical activity, n (%)			
No	7,554 (66.1)	3,870 (33.9)	< 0.001
Yes	3,897 (70.4)	1,635 (29.6)	

Self-rated health, n (%)			
Excellent	664 (78.9)	178 (21.1)	< 0.001
Good	3,947 (75.5)	1,278 (24.5)	
Fair	5,140 (69.1)	2,301 (30.9)	
Poor	1,437 (49.1)	1,492 (50.9)	
Very poor	179 (44.3)	225 (55.7)	

BMI (kg/m^2^), mean (SD)	22.9 (2.8)	24.1 (3.2)	< 0.001

*t*-test or chi-square test as appropriate

### Prevalence of hypertension by SRH

**Figure 1:**
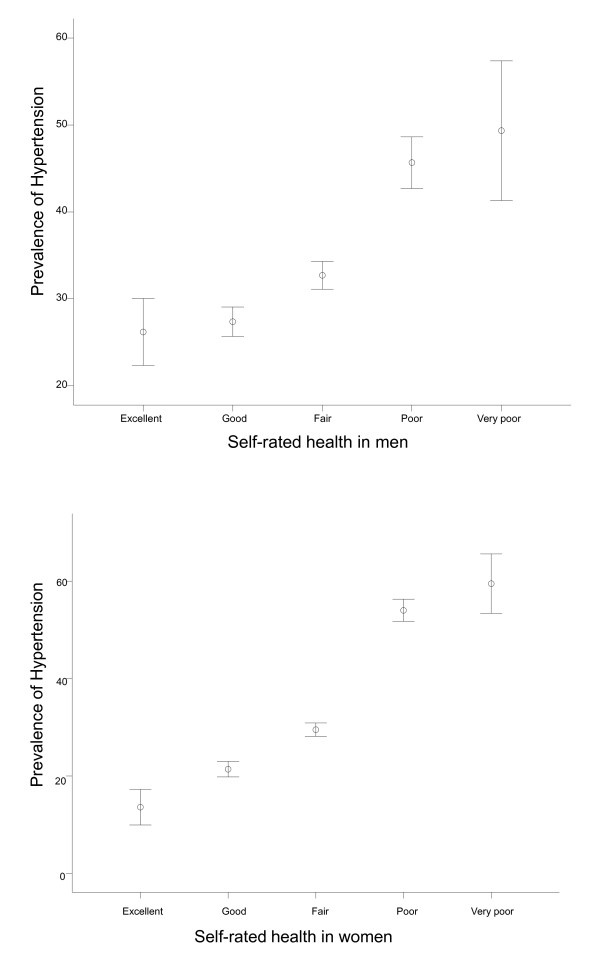
**The prevalence of hypertension by self-rated health in men and women**.

The prevalence of hypertension for each level of SRH was 21.1% (excellent), 24.5% (good), 30.9% (fair), 50.9% (poor) and 55.7% (very poor) (Figure 1). People with poorer SRH had higher prevalence of hypertension (*p *< 0.001).

### SRH by gender and age

The distribution of SRH was different between men and women (*p *< 0.001), and women were more likely to rate their health status poorer than men. Both men and women who were older tended to rate their SRH as poorer than the younger subjects (*p *< 0.001; Table 2).

**Table 2:** Distribution of self-rated health (SRH) by gender and age

	SRH				
	
	Excellent	Good	Fair	Poor	Very poor
Men					
Age (yrs)					
20-39	190 (9.8)	853 (43.8)	795 (40.8)	96 (4.9)	13 (0.7)
40-59	222 (7.6)	1,066 (36.6)	1,310 (45.0)	284 (9.8)	29 (1.0)
60 ≤	111 (3.7)	799 (26.9)	1,240 (41.7)	714 (24.0)	110 (3.7)

Women					
Age (yrs)					
20-39	155 (7.5)	854 (41.5)	934 (45.3)	107 (5.2)	10 (0.5)
40-59	140 (4.6)	1,027 (33.5)	1,497 (48.8)	370 (12.1)	36 (1.2)
60 ≤	50 (1.2)	709 (17.1)	1,802 (43.4)	1,382 (33.3)	212 (5.1)

Values are number (%)

Trend test for age by SRH in both men and women: *p *< 0.001

Chi-square test for gender by SRH: *p *< 0.001

### SRH and hypertension--logistic regression models

Results of logistic regression analyses are summarized in Table 3. The unadjusted odds ratio (OR) of participants rating their SRH as very poor for hypertension in men was 2.75 (95% CI 1.89-3.99) compared with the reference group of participants rating SRH as excellent and that in women was 9.34 (95% CI 6.26-13.93). SRH was independently associated with hypertension in men (OR: 1.70; 95% CI: 1.13-2.58) and women (OR: 2.83; 95% CI 1.80-4.44) after adjustment for age, marital status, education, smoking status, alcohol consumption, physical activity and BMI. Adjusted SRH by gender interaction was statistically significant (*p *< 0.001).

**Table 3:** Logistic regression models stratified by gender for the association between self-rated health (SRH) and hypertension

	SRH				
	
	Excellent	Good	Fair	Poor	Very poor
	(n = 842)	(n = 5,225)	(n = 7,441)	(n = 2,929)	(n = 404)
Men					
Unadjusted	1.00	1.06(0.86-1.32)	1.37(1.11-1.69)	2.37(1.88-2.99)	2.75(1.89-3.99)
(1) Adjusted for age	1.00	0.95(0.76-1.19)	1.10(0.88-1.36)	1.40(1.10-1.79)	1.49(1.01-2.20)
(2) Model 1 plus marital status and education	1.00	0.95(0.76-1.18)	1.10(0.88-1.38)	1.42(1.11-1.81)	1.50(1.01-2.22)
(3) Model 2 plus smoking status and alcohol drinking	1.00	0.92(0.73-1.15)	1.08(0.87-1.36)	1.37(1.07-1.76)	1.50(1.00-2.24)
(4) Model 3 plus physical activity	1.00	0.93(0.74-1.16)	1.09(0.87-1.37)	1.38(1.08-1.78)	1.51(1.01-2.26)
(5) Model 4 plus BMI	1.00	0.89(0.71-1.12)	1.03(0.82-1.29)	1.38(1.07-1.78)	1.70(1.13-2.58)

Women					
Unadjusted	1.00	1.73(1.25-2.39)	2.66(1.94-3.65)	7.46(5.40-10.32)	9.34(6.26-13.93)
(1) Adjusted for age	1.00	1.28(0.91-1.81)	1.48(1.05-2.07)	2.58(1.83-3.65)	2.87(1.88-4.40)
(2) Model 1 plus marital status and education	1.00	1.19(0.84-1.69)	1.34(0.95-1.88)	2.33(1.64-3.30)	2.61(1.70-4.00)
(3) Model 2 plus smoking status and alcohol drinking	1.00	1.24(0.87-1.77)	1.37(0.96-1.94)	2.38(1.66-3.40)	2.76(1.78-4.29)
(4) Model 3 plus physical activity	1.00	1.25(0.87-1.78)	1.39(0.98-1.97)	2.44(1.70-3.49)	2.87(1.84-4.46)
(5) Model 4 plus BMI	1.00	1.28(0.89-1.83)	1.38(0.97-1.97)	2.36(1.64-3.40)	2.83(1.80-4.44)

Odds ratios (OR) with 95% confidence intervals

Test for SRH by gender interaction term in the model adjusted for SRH, gender, age, marital status, education, smoking status, alcohol drinking, physical activity and BMI: *p *< 0.001

## Discussion

In this study of a community dwelling Korean adult population, SRH was independently associated with hypertension after controlling for other related factors such as age, gender, marital status, education, smoking status, alcohol consumption, physical activity and BMI. Further, there were significant gender differences in the association between SRH and hypertension. SRH was more strongly associated with hypertension in women, compared to men.

Our findings are similar to the results from previous studies suggesting an association between SRH and morbidity or mortality [1-8]. According to the study, there was a dose-response relationship, which means that the prevalence of hypertension was highest for the category of very poor SRH and less for fair SRH. However, the mechanisms involved in this relation are still not clear. SRH is a summary statement concerning the ways in which various aspects of health are combined together [21]. SRH involves subjective as well as objective measures of health and the reliability of SRH has been shown to be high [22]. Recent studies have attempted to examine the possibility that SRH has a biological basis. While SRH may be associated with inflammatory cytokines in the elderly population, humoral immune markers may be more sensitive to poor SRH in healthy individuals [9-11]. In addition, the ways of judging their health status may vary according to gender, age groups, and different social and cultural backgrounds [23]. Since most studies have been performed in Western populations, this study may contribute to the understanding of SRH in Asian populations. In our study, the distribution of SRH was different between men and women: women were more likely to rate their health status poorer than men. Furthermore, both men and women who were older rated their SRH as poorer than the younger subjects.

Although several studies revealed that the effect of SRH on the prediction of mortality seems to be more apparent for men than women, little is known about gender differences in the association between SRH and hypertension [1,14,15]. While men are likely to rate their health mainly by comparing their health status with the health of other men, women tend to rate their health by considering various sources including the health status of themselves and their family. Men suffer more from life-threatening conditions than women do, but women suffer more from chronic, disabling conditions [24-26]. Furthermore, while men tend to reflect mainly serious and life-threatening disease in their assessment of SRH, women tend to reflect both life-threatening and non-life-threatening diseases [19]. Therefore, poor ratings of health by men may reflect more serious conditions, but those by women may imply more chronic conditions. Women are more likely to include mild diseases in their general health assessment than men. In our study, SRH was more strongly associated with hypertension in women than in men. As hypertension is considered as one of typical examples of chronic disease, the finding may be understood in this context.

This study has several limitations. First, the study was a population-based cross-sectional survey. With this kind of study design, it is often not possible to establish a temporal relationship. To confirm this association, a prospective study needs to be done. Second, there is a possibility of reverse causality; SRH could be not the cause but the outcome for hypertension, because hypertension labeling may adversely affect SRH. Hypertension is an asymptomatic condition that affects 30% or more adults [27] and most hypertensive individuals remain unaware of their condition [28]. Since labeled hypertensives are more likely to report poorer SRH than normotensives, the result of this study should be interpreted in caution [12,13,29]. Nevertheless, considering SRH is a proven predictor of mortality and it has been examined as an objective measure of health as well as a subjective measure, SRH may also be considered to be a risk factor for hypertension. Third, the relatively low response rate (41.1%) of invited participants may introduce response bias. However, this is the first report on the association of SRH with hypertension in a Korean adult population. The sample was representative of the source population using the national registration records. Furthermore, information on socio-demographic factors (age, gender, marital status, education) and health behaviors (smoking status, alcohol consumption, physical activity) were collected, and BMI and blood pressure were measured. The related factors were adjusted in logistic regression models. Previous studies have reported SRH as a predictor of mortality mostly in Western populations, but this study revealed the association between SRH and hypertension in an Asian population. In addition, although some studies dealt with SRH as an outcome of hypertension and there was no report on gender differences, this study showed the possibility of SRH as a predictor of hypertension and this association was modified by gender.

## Conclusions

In conclusion, SRH was found to be independently associated with hypertension in men (OR: 1.70; 95% CI: 1.13-2.58) and women (OR: 2.83; 95% CI 1.80-4.44) after adjustment for other related factors. Women were more likely to rate their health status poorer than men, and SRH was more strongly associated with hypertension in women than in men. Further research is needed to confirm the association in a prospective manner.

## Competing interests

The authors declare that they have no competing interests.

## Authors' contributions

HYS and MHS designed the study and performed the statistical analyses. HYS, MHS, and JAR interpreted the results. HYS and MHS drafted the manuscript. All authors read and approved the final manuscript.

## Pre-publication history

The pre-publication history for this paper can be accessed here:

http://www.biomedcentral.com/1471-2458/12/135/prepub
